# Evaluation of an intensive, private, community, upper limb rehabilitation program for people with chronic stroke: a mixed methods case series

**DOI:** 10.3389/fresc.2026.1809550

**Published:** 2026-06-12

**Authors:** Alana Spirou, Ines Serrada, Kate Smedley, Julian Sartoretto, Brenton Hordacre

**Affiliations:** 1 Innovation, IMPlementation and Clinical Translation in Health (IIMPACT in Health), College of Health, Adelaide University, Adelaide, SA, Australia; 2 ONE Rehabilitation Service, Ashford, SA, Australia

**Keywords:** case series, chronic stroke, occupational therapy, physiotherapy, stroke rehabilitation, upper limb

## Abstract

**Purpose:**

Increased rehabilitation intensity may improve upper-limb recovery post-stroke. This study explored early preliminary evidence of effectiveness and participant experiences of a private, intensive, community, upper-limb rehabilitation program (Neuroboost).

**Methods:**

Pre-post case series using a concurrent mixed methods design. People with chronic stroke and upper limb impairment enrolled in a six-week, 18-hour, rehabilitation program (Neuroboost) were invited to participate. Quantitative measures (pre-post) investigated upper-limb impairment/activity (Fugl-Meyer Assessment Upper Extremity – FMA-UE; Action Research Arm Test – ARAT), self-efficacy (Stroke Self-Efficacy Questionnaire – SSEQ), and quality of life (EuroQol-5 Dimension Questionnaire – EQ-5D-5L; EuroQol Visual Analogue Scale – EQ-VAS). Semi-structed interviews were conducted within four-weeks of Neuroboost completion.

**Results:**

Two participants were recruited (both female, 48 and 53 years). FMA-UE improved in both participants (3 and 6 points). ARAT (0 and 3 points), SSEQ (11 and −15 points), EQ-5D-5L (−0.01 and −0.29), and EQ-VAS (0 and 55 points) changes were variable. Participants reported themes of Commitment, Social, Technology, Arm Movement, Quality of Life, Expectations, and Environment.

**Conclusion:**

Preliminary findings from a small sample suggest that delivering intensive rehabilitation in a private setting may promote upper-limb recovery in chronic stroke survivors. A larger study appears worthy to establish evidence of effectiveness.

## 1 Introduction

Stroke is a leading cause of adult disability (1). Two-thirds of stroke survivors experience persistent upper limb impairments, affecting quality of life and self-efficacy (2, 3). Most recovery occurs in the initial weeks after stroke, likely coinciding with a period of heightened neuroplasticity (4). However, recovery remains possible months to years after stroke but requires large amounts of practice (5, 6). For example, an intensive trial demonstrated 90 h of rehabilitation over 5 weeks led to large and meaningful gains in upper limb impairment and activity compared to usual care (6). In support, systematic reviews show a positive relationship between increased hours of rehabilitation and reduction of impairments (7–10). An increase of 240% beyond usual care has been proposed to drive meaningful recovery (11).

Despite strong evidence for increased amount of rehabilitation, low levels of therapy persist in the community setting (12). Survey data suggests that current community stroke services are insufficient, delivering one to two weekly sessions of 30-minute duration (12). Previous research has not investigated the pragmatic implementation of intensive upper limb rehabilitation into a private community setting. Therefore, it is unclear whether delivering large amounts of therapy in the community is in fact possible or if it leads to better outcomes for people with chronic stroke.

Neuroboost is an intensive upper limb rehabilitation program offered by One Rehabilitation Service; a private neurological interdisciplinary therapy clinic located in Adelaide, South Australia. The six-week program provides three one-hour sessions per week, totalling 18 hours of upper limb therapy, equating to a 300-600% increase over usual private community services. The aim of this study was to explore early preliminary evidence of effectiveness and investigate participant experiences of Neuroboost. It was hypothesised that Neuroboost would lead to improved recovery with positive participant experiences.

## 2 Methods

This study was reported using the Mixed Methods Reporting in Rehabilitation and Health Sciences and the CARE Case Report Guidelines (Supplementary Material 1). Ethics approval was obtained (UniSA Human Research Ethics Committee, ID: 206332). The protocol was pre-registered on Open Science Framework (October 2024). Participants provided written consent.

### 2.1 Design

**Figure 1:**
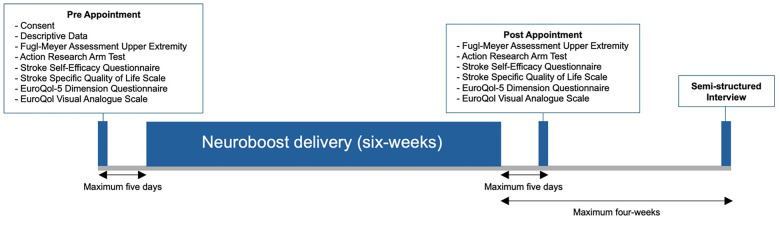
Study procedures.

A pre-post case series using a concurrent mixed methods design evaluated Neuroboost (Figure 1). A case series was selected as it allows for investigation into emerging interventions for a specific population (13). Quantitative pre-post data described changes in upper limb function, self-efficacy, and quality of life. A qualitative descriptive approach was integrated to investigate the participant experience, and capture any specific changes in upper limb function, participation, or quality of life that quantitative measures cannot describe (14).

### 2.2 Participants

People enrolled in Neuroboost (purposive sampling) between October 2024 and March 2025 were invited to participate. Neuroboost inclusion criteria, determined by One Rehabilitation, required people to have an upper limb impairment resulting from any neurological disorder, and adequate cognition to complete rehabilitation in a group setting. For the purpose of this study, we further limited inclusion to; people aged 18 years and over, with chronic stroke [defined as >6 months post-stroke (15)], a documented level of upper limb impairment [Fugl-Meyer Upper-Extremity (FMA-UE) between 15–60/66], intact cognition (≥24/30 on Mini-Mental State Examination), and no Medical co-morbidities preventing participation in intensive rehabilitation.

### 2.3 Neuroboost

Neuroboost was a six-week, semi-supervised (2:1 patient to therapist), program that targeted fine and gross motor upper limb skills. Participants attended three one-hour in-clinic sessions per week with an experienced neurological physiotherapist or occupational therapist (total 18 therapy sessions). Participants were prescribed a home exercise program to be completed for 30-60 minutes on four days per week. Home exercises were provided through Physitrack and compliance was recorded via self-report. Overall, Neuroboost aimed to deliver 18 hours of in-clinic therapy and encouraged 12-24 hours of home exercise over six-weeks.

Neuroboost was a structured, principle-driven upper limb rehabilitation program rather than a fixed set of prescribed exercises. The reproducible component of Neuroboost lies in the systematic application of high-repetition, task-specific, and goal-directed practice guided by motor learning. Neuroboost was underpinned by motor learning principles including intensive repetition, salience, progressive challenge, and active engagement (16, 17). Therapists aimed to maximise active practice time and encouraged use of the affected limb while minimising compensatory strategies through verbal and visual feedback. Therapy content was individualised according to participant impairments, movement capacity, fatigue levels, and rehabilitation goals. For example, participants with predominantly proximal movement capacity focused on shoulder and elbow activation, supported reaching, and initiation of wrist and hand movement, whereas participants with greater distal control progressed toward active wrist movement, grasp-release activities, object manipulation, and fine motor coordination tasks. Task difficulty was progressively increased by modifying movement speed, accuracy demands, resistance, range of motion, cognitive load, and task complexity once activities could be completed with minimal error or compensation.

By way of example, representative impairment-focused exercises included shoulder external rotation tasks performed toward visual targets (e.g., markers positioned on an overbed table), rolling a Pilates ball to encourage controlled upper limb movement, and technology-assisted reaching activities. Wrist extension training involved lifting the dorsum of the hand toward visual targets, with progression achieved by increasing target height, adding hand weights, or incorporating game-based technology tasks. Early thumb activation and abduction exercises were facilitated using visual cueing strategies such as separating playing cards positioned between the thumb and index finger. Representative functional task-specific activities included reaching to targets positioned at varying distances and heights, grasp and release practice using objects of different sizes and weights, cone stacking, card flipping, utensil use, folding clothes, tissue pulling and tearing activities, and fine motor coordination tasks such as beading and threading. Many of these activities could also be completed using gamified technology-assisted formats through systems such as Pablo, ReJoyce, and MusicGlove. Technology offered benefits of augmented visual feedback, increased engagement, supported gamified practice, and encouraging a high number of movement repetitions during therapy.

### 2.4 Quantitative data collection

Age, sex, time since stroke, affected limb, pre-stroke hand dominance, and rehabilitation history were collected. Clinical outcomes included FMA-UE, action research arm test (ARAT), Stroke Self-Efficacy Questionnaire (SSEQ), and EuroQol-5 Dimension (EQ-5D-5L). Psychometric properties are reported in Supplementary Material 2. Baseline and post-treatment measures were obtained within 5 days of beginning, and 5 days of completing Neuroboost respectively. Measures were obtained by a trained investigator not associated with One Rehabilitation or intervention delivery.

### 2.5 Qualitative data collection

Within four-weeks of Neuroboost completion, individual semi-structured interviews were conducted. Interview questions explored overall Neuroboost experience, and subjective changes in upper limb function, participation, and quality of life (see Supplementary Material 3 for interview guide). Interviews were 16-22 minutes in duration, conducted over the phone or via Zoom by a trained researcher, and audio-recorded. Credibility was maintained through researcher triangulation to determine themes that captured the range and depth of participant responses. Member checking was employed to increase credibility, ensuring participant opinions were represented correctly (18). Participants were emailed their transcript to allow for amendment of information prior to data analysis. Confirmability was addressed through researcher triangulation and member checking, as well as using direct quotes. Dependability was demonstrated through audio-recorded interviews, transcription verbatim, and independent coding checks by two researchers (AS and IS). Credibility, confirmability, and dependability were further addressed through a pilot interview on one person with stroke. The pilot interview ensured clarity and relevance of questions and allowed for refinement of the interview guide (19). Detailed and contextual information about study methods and participants addressed transferability. Reflexivity was maintained through regular peer-debrief meetings.

### 2.6 Data analysis

#### 2.6.1 Quantitative

Australian preference weights were used to convert EQ-5D-5L results to a single utility score (20). Change values (post – pre scores) were calculated for FMA-UE, ARAT, SSEQ, EQ-5D-5L utility score, and EQ-VAS. Change values were compared to the minimal clinically important difference (MCID) of each outcome measure (see Supplementary Material 2). Statistical tests were deemed inappropriate due to the small sample size.

#### 2.6.2 Qualitative

Interviews were recorded and transcribed verbatim and analysed with an inductive thematic analysis (21, 22). First, two researchers individually divided the text into meaning units. Each meaning unit was independently labelled with a code that summarised the meaning of the data. After initial coding, researchers met to ensure consensus between codes. Next, researchers independently organised codes into categories and themes, where impressions of the data were regularly discussed to achieve agreement.

## 3 Results

### 3.1 Participant clinical presentation and rehabilitation profile

Two people with stroke were recruited (Table 1). Both participants received four months of inpatient rehabilitation, followed by public outpatient and community physiotherapy and occupational therapy services. Once discharged, participants began private rehabilitation at One Rehabilitation Service. Both presented with chronic, moderate upper limb impairment following left hemispheric stroke, with similar time since stroke (P01: 41 months; P02: 34 months). P01 demonstrated greater impairment of distal upper limb function, with minimal active wrist and hand movement at baseline (FMA-UE wrist = 0/10, hand = 2/14), and very limited functional grasp or object manipulation (ARAT = 3/57). Proximal movement was relatively better preserved, suggesting primary deficits in distal motor control and fine motor function. P02 presented with a more balanced impairment profile, with some preserved wrist movement (FMA-UE wrist = 2/10) and greater baseline functional capacity (ARAT = 15/57), including partial grasp and grip ability. Despite this, distal dexterity and coordination remained limited. These differences in baseline motor capacity informed task selection and progression during Neuroboost, with both participants receiving predominantly task-specific training, but with greater emphasis on proximal activation and movement initiation for P01, and relatively greater emphasis on wrist and hand control for P02.

**Table 1:** Participant demographics (*n* = 2). .

Participant Code	Age	Sex	Time Since Stroke (Months)	Affected Limb	Pre-stroke Hand Dominance
P01	48	Female	41	Left	Right
P02	53	Female	34	Left	Right

### 3.2 Program delivery, adherence and clinical outcomes

At time of data collection, an example of client cost for a Neuroboost session was half the standard occupational therapy National Disability Insurance Scheme hourly rate (AUD $193.99/2 = AUD $96.99 per session), as all sessions were conducted with a 2:1 patient to therapist ratio (23). Therefore, an example of the minimum total client cost for Neuroboost was AUD $2133.89, including pre/post assessments and therapy. Both participants completed the full 18 hours of in-clinic therapy. Self-report of home exercise (Physitrack) indicated low adherence, where P01 and P02 completed 41.6% and 0% of prescribed exercise, respectively.

Quantitative outcomes are summarised in Table 2. Both participants demonstrated small improvements in upper limb impairment (FMA-UE), with changes of 3 points (P01) and 6 points (P02), respectively. Only P02 exceeded the minimal clinically important difference for this measure. Changes in upper limb activity (ARAT) were minimal, with no change observed in P01 and a 3-point improvement in P02. Self-efficacy outcomes were variable between participants. P01 demonstrating an increase in SSEQ (change +11), while P02 demonstrated a decrease (change −15). Quality of life showed small and inconsistent changes, with minimal change in EQ-5D-5L utility scores (P01: −0.01; P02: −0.29) and variable changes in EQ-VAS scores (P01: 0; P02: +55).

**Table 2:** Pre-post neuroboost results and change values for each outcome measure and relevant subsections.

Outcome Measure	P01			P02		
	Pre	Post	Change Value	Pre	Post	Change Value
FMA-UE Total (/66)	17	20	3	21	27	**6** *
Arm (/36)	15	16	1	16	20	4
Wrist (/10)	0	0	0	2	4	2
Hand (/14)	2	4	2	3	3	0
Coordination (/6)	0	0	0	0	0	0
ARAT Total (/57)	3	3	0	15	18	3
Grasp (/18)	0	0	0	11	12	1
Grip (/12)	0	0	0	1	3	2
Pinch (/18)	0	0	0	0	0	0
Gross Movement (/9)	3	3	0	3	3	0
SSEQ Total (/130)	69	80	**11** *	50	35	−15
EQ-5D-5L Utility Score	0.48	0.47	−0.01	0.05	−0.24	−0.29
EQ-VAS Total	80	80	0	20	75	**55** *

* Change values exceeding the MCID are shown in bold with an asterisk.

FMA-UE, Fugl-Meyer Assessment Upper Extremity; ARAT, Action Research Arm Test; SSEQ, Stroke Self-Efficacy Questionnaire; EQ-5D-5L, EuroQol-5 Dimension Questionnaire; EQ-VAS, EuroQol Visual Analogue Scale; MCID, minimum clinically important difference.

### 3.3 Participant experiences

Thematic analysis revealed seven themes of *Commitment, Social, Technology, Arm Movement, Quality of Life, Expectations,* and *Environment* (Table 3)*. Commitment* was the largest theme, divided into three categories of Intensity, Home Exercise, and Balancing Life and Therapy. Differing opinions existed within themes.

**Table 3:** Examples of quotes, codes, categories and themes. .

Quote	Code	Category	Theme
*“Yeah, I thought the commitment was uh quite easy to commit to and set up a routine.”* (P01)	Easy to commit and set up a routine	Intensity	*Commitment*
*“I thought they [home exercises] were, um quite doable, that it wasn't an overcommitment.”* (P01)	Doable home exercises, not an overcommitment	Home Exercise	
*“Don't overcommit yourself and when you're going to do the program, just focus strictly on the program without doing anything else on top.”* (P02)	Don't overcommit yourself, focus strictly on the program	Balancing Life and Therapy	
**Quote**	**Code**		**Theme**
*“Yep, uh so um it's quite a close knit two-person group. So, um, it um, it sort of made you interact with the other person.”* (P01)	Close knit two-person group made you interact with the other person		*Social*
*“Um, I really enjoyed that [technology] part of it. I think it made— it makes you um you, you're doing sort of it, you feel like you're doing involuntary movements.”* (P01)	Enjoyed technology, you feel like you’re doing involuntary movements		*Technology*
*“And just the way my hand has been sitting lately, it's slightly open.”* (P01)	My hand has been sitting slightly open lately		*Arm Movement*
*“I'm using my hands to try and do some artwork at home just to try and— as a form of well-being for me.”* (P02)	Using my hands to do artwork as a form of well-being		*Quality of Life*
*“Um, overall, my experience um was uh a good experience.”* (P01)	Overall a good experience		*Expectations*
*“Could be a little bit bigger, I think the room, I think so yes.”* (P02)	Room could be bigger		*Environment*

#### 3.3.1 Theme 1: commitment

##### 3.3.1.1 Intensity

Participant one found the three one-hour sessions per week “*easy to commit to”* and recognised the importance of intensive training.

“Me in this situation I realised that you need the repetitiveness… I understand the over six-week program was needed.” (P01)

Conversely, P02 described Neuroboost as “*very intensive,”* resulting in mental and physical fatigue. They suggested a *“four-week”* program rather than six-weeks.

“I got burnout [during Neuroboost]… that's what really affected me both mentally and physically.” (P02)

##### 3.3.1.2 Home exercise

Home exercise requirements were manageable for P01, as practice was recommended only on the alternate days of Neuroboost. Participant two believed home exercises were prescribed well although had difficulty committing due to a lack of time.

“I thought they [home exercises] were quite doable, that it wasn't an overcommitment.” (P01)

“I didn't have enough chance to do the home program… it's just trying to find the time to do it… you gotta have the want and the time.” (P02)

Overall, participants understood the importance of home exercise as a part of rehabilitation.

“To get something out of Neuroboost… you've gotta commit to it at home.” (P01)

“It's all about doing the homework.” (P02)

##### 3.3.1.3 Balancing life and therapy

For P02, participation prior to Christmas impacted commitment due to competing life priorities. Participant one agreed intensive rehabilitation would be harder to commit to when managing obligations such as work.

“It's just too busy at the end of the year with everything going on with life and Christmas… pick a timeframe that's going to suit you… without overcommitting to other things.” (P02)

“How I would commit to that [Neuroboost] around a work situation complicates things.” (P01)

#### 3.3.2 Theme 2: *social*

Participants described the positives of completing Neuroboost with another client. They found value in learning from each other and sharing personal updates during sessions, encouraging social interaction and support.

“It's quite a close knit two-person group… it sort of made you interact with the other person.” (P01)

“It's always nice to spend a few minutes just having a bit of an update with how things are going in their [other participant's] life… there's a small percentage of us it's always nice to connect.” (P02)

Contrastingly, this could be unfavourable as P01 described talkative partners as distracting. The group needed to be “*monitored by the therapist”* to be advantageous*.* An ideal group number was suggested to be “*three to four people.”*

“It [talkative participant] was quite draining… don't take away my fun part of the time [using technology] by distracting me with questions.” (P01)

#### 3.3.3 Theme 3: technology

Technology was described as fun and enjoyable. Participant one stated that technology made Neuroboost more engaging and achievable. Technology that combined visual tasks with upper limb therapy was beneficial for those with visual impairment.

“I really enjoyed that [technology] part of it… they’re sort of a bit more of a fun aspect.” (P01)

“I did like the tech side of it, that was quite good… I liked doing the driving [games]… that helped me try and see things with my visual impairment.” (P02)

Participants also addressed the limited variety. Technology was described as “*old school”* by P02 and occupational therapy based by P01, where more desktop devices would improve options.

“I think the technology is a little bit old school… it could do with a bit of updating.” (P02)

“It was more OT [occupational therapy] equipment [technology] based, I think there should be more desktop digital equipment.” (P01)

#### 3.3.4 Theme 4: Arm movement

Participant one reported increased attention towards their affected arm, leading to more “*shoulder and hand”* movement. The resting position of their hand has been sitting *“slightly open,”* allowing attempts at gripping objects.

“I'm just giving it [affected arm] more attention… I’m just trying to move it naturally, rather than using this [unaffected] hand.” (P01)

Participant two did not perceive any large changes in arm movement but rather found that Neuroboost helped stimulate “*touch, feel, and sensation of the hand.”* They noted that focusing on arm therapy for six weeks had a negative impact on mobility.

“Nothing has improved dramatically [arm movement]… there's no such miracle type thing that has happened.” (P02)

#### 3.3.5 Theme 5: quality of life

Quality of life remained unchanged after Neuroboost. Participants reported no difference in personal roles, family roles, or engagement in hobbies.

“I think it's [quality of life] pretty much the same but I'm still trying to participate in everything that I can.” (P02)

“I didn't think that [quality of life] was a, a problem before, so no, there's been no difference.” (P01)

However, P01 highlighted a meaningful shift in outlook towards arm therapy and improved self-efficacy. Internal motivation to persist with arm therapy increased and was accompanied by a sense of optimism about regaining arm function.

“My outlook on me wanting to persist with arm therapy and being able to succeed in getting arm movement back has, I guess, increased.” (P01)

#### 3.3.6 Theme 6: expectations

Participants stated Neuroboost was “*nice to do”* and “*overall a good experience.”* Participant one desired “*more physio input”* as sessions were “*occupational therapy based.”* Participant two emphasised that therapists would end sessions 10-15 minutes early to complete documentation, which was perceived as “*not right.”*

“They [therapists] do say it is one hour [per session] but to be quite honest it's not an hour they give you.” (P02)

Furthermore, P02 mentioned an extra therapist would improve time efficiency as sessions sometimes felt disorganised. They suggested a structured session program that aligned with home exercises and provided education on upper limb impairments.

“A bit of a program or something about what we would be doing each week so then we can focus maybe on some home activities that might address what we're going to be learning that day.” (P02)

#### 3.3.7 Theme 7: environment

Participants believed that the Neuroboost room could have been “*bigger.”* A larger space would permit simultaneous access to technology and allow for easier transitions between areas of the room. Participant one acknowledged that therapists did well at “*making the most of the small office space.”*

“I think if it was a bigger space… they could offer more to the second person as far as [technology] equipment.” (P01)

“Could do to be a little bit bigger [the room]… when you have to move from one [area] to another to make it a bit of a quicker process.” (P02)

## 4 Discussion

This study evaluated early preliminary evidence of effectiveness of Neuroboost on clinical outcomes in people with chronic stroke, and participant experiences of this program. There was some signal to suggest improved upper limb outcomes. Neuroboost positively impacted self-efficacy in one participant; however, had a conflicting influence on quality of life. Commitment to intensive rehabilitation was challenging for one person. Participants highlighted features of Neuroboost that increased enjoyment, such as technology and the social interaction of group programs. Potential improvements may include increased interdisciplinary input, providing session structure, enhancing room space, and offering more therapeutic technology.

Quantitative data provided signal to suggest improved upper limb outcomes, although participant perspectives did not consistently align. Participant two achieved a clinically meaningful improvement in upper limb impairment (FMA-UE 6 points); however, this participant self-reported minimal change in arm movement. Previous qualitative research suggests there is often a gap between young stroke survivors (aged <64 years) expectations of recovery and reality, providing one possible explanation for this discrepancy (24). Conversely, P01 perceived an increase in shoulder and hand movement despite a smaller FMA-UE change (3 points). Interestingly, majority of improvement for P01 was in the FMA-UE hand subsection, whereas P02 achieved most change in the arm subsection. Changes in hand impairment may be more noticeable than proximal upper limb changes, as hand movement is essential for functional activities such as grasping objects (25). Furthermore, there was indication to suggest arm activity improved in one participant (ARAT 3 points). Upper limb improvements in this clinical setting were smaller than that of previous intensive chronic stroke trials, achieving gains of 10.2 on FMA-UE and 7.3 on the ARAT (6). Differences in the magnitude of improvement are likely due to the amount of practice, where 90 h of training was provided in the past trial (6), but 18 h here. Of course, it should be noted that usual care in the earlier trial (30-60 minutes per week for 5 weeks) led to negligible changes (6), providing some further support that Neuroboost is an advance on usual care. Neuroboost data provides the first indication that intensive rehabilitation in a real-life private setting may promote greater upper limb recovery.

Self-efficacy improved in one participant (P01), indicated by a meaningful SSEQ increase of 11 points (26). Previous research found a smaller, but statistically significant mean SSEQ increase of 4.9 points after 90 hours of upper limb therapy (6). Qualitative insights supported quantitative data as P01 expressed optimism and confidence about achieving upper limb recovery through future rehabilitation. Qualitative change in arm movement for P01 may have provided direct evidence of personal capability, contributing to enhanced belief in the ability to achieve future recovery goals. Improvement in self-efficacy is an important finding as self-efficacy levels are positively related to rehabilitation outcomes and treatment adherence (27). However, an unanticipated result was a 15-point SSEQ reduction for P02. It is uncertain why self-efficacy decreased in this participant, but two possibilities include increased fatigue with greater rehabilitation intensity (28), or the perception of minimal upper limb changes possibly leading to doubt in recovery potential.

Both participants completed all Neuroboost sessions (18 hours). P01 found the intensity requirements to be feasible while P02 reported difficulty with commitment. Rehabilitation history, personal variables, and differences in expectations may influence ability to tolerate intensive rehabilitation. Therefore, increased education to prepare participants for the large intensity commitment should be incorporated. Despite participants understanding the importance of home exercise, commitment was below target (41.6% and 0% self-reported completion rates). Likewise, home exercise adherence among community-dwelling stroke survivors is reportedly low (<50%) in the literature (29, 30). Strategies to improve home exercise compliance are needed. Moreover, participants agreed that Neuroboost is a more cost-effective way to access therapy with a qualified clinician rather than a standard 1:1 appointment. Group rehabilitation has promise, providing both social and financial benefits for participants, resource efficiencies for private clinics, further supported by evidence of improved clinical outcomes in the wider literature (31).

### 4.1 Limitations

As Neuroboost was delivered as a pragmatic clinical service rather than a protocolised research intervention, detailed session-by-session exercise logs were not routinely collected. Furthermore, the sample size was small, reducing reliability and trustworthiness of results. There were challenges with recruitment, since Neuroboost was a new program with a financial commitment for participants, and recruitment occurred over the Christmas holiday period. Furthermore, it is unclear if potential gains extended beyond program completion as long-term outcomes were not obtained. There was some indication that upper limb benefits persisted as P01 described positive changes in arm movement during the interview (four weeks after Neuroboost completion). Our data should be viewed as a preliminary signal to indicate possible effects of Neuroboost.

### 4.2 Future recommendations

Early positive signals of Neuroboost suggest intensive upper limb rehabilitation in a private community setting may have merit. A larger study appears warranted to further demonstrate clinical effectiveness of Neuroboost. Slow participant recruitment should be anticipated, with adequate time for data collection allowed. Rigour could be increased through an alternative research design, such as a waitlist control trial.

## 5 Conclusion

This study makes a unique contribution to the literature as it is the first to evaluate a private clinical service offering intensive upper limb rehabilitation in the community. There is signal to suggest intensive rehabilitation might promote improved upper limb outcomes in people with chronic stroke when delivered in real-life clinical settings. Neuroboost may have influenced self-efficacy and quality of life outcomes; however, a larger study with increased rigour is required to confirm clinical effectiveness.

## Data Availability

The original contributions presented in the study are included in the article/Supplementary Material, further inquiries can be directed to the corresponding author.
