# Supplementary material for: Evaluation of an intensive, private, community, upper limb rehabilitation program for people with chronic stroke: a mixed methods case series
Source: Front Rehabil Sci. 2026 Jun 12;7:1809550. doi: 10.3389/fresc.2026.1809550 (PMC13303582; doi:10.3389/fresc.2026.1809550)
Supplement: Supplementary file 1 [file Supplementaryfile1.pdf]

Supplementary Material

Appendix 1: CARE Case Report Guidelines

CARE case report guidelines

CARE Checklist of information to include when writing a case report

BY-NC-ND

Topic	Item	Checklist item description	Reported on Line
Title	1	The diagnosis or intervention of primary focus followed by the words "case report"	Title
Key Words	2	2 to 5 key words that identify diagnoses or interventions in this case report, including "case report"	Keywords
Abstract (no references)	3a	Introduction: What is unique about this case and what does it add to the scientific literature?	Abstract
	3b	Main symptoms and/or important clinical findings	Abstract
	3c	The main diagnoses, therapeutic interventions, and outcomes	Abstract
	3d	Conclusion—What is the main "take-away" lesson(s) from this case?	Abstract
Introduction	4	One or two paragraphs summarizing why this case is unique (**may include references**)	Introduction
Patient Information	5a	De-identified patient specific information.	Results, section 3.1
	5b	Primary concerns and symptoms of the patient.	Results, section 3.1
	5c	Medical, family, and psycho-social history including relevant genetic information	Results
	5d	Relevant past interventions with outcomes	Results
Clinical Findings	6	Describe significant physical examination (PE) and important clinical findings.	Results
Timeline	7	Historical and current information from this episode of care organized as a timeline	N/A
Diagnostic Assessment	8a	Diagnostic testing (such as PE, laboratory testing, imaging, surveys).	N/A
	8b	Diagnostic challenges (such as access to testing, financial, or cultural)	N/A
	8c	Diagnosis (including other diagnoses considered)	Results
	8d	Prognosis (such as staging in oncology) where applicable	N/A
Therapeutic Intervention	9a	Types of therapeutic intervention (such as pharmacologic, surgical, preventive, self-care)	Methods, section 2.3
	9b	Administration of therapeutic intervention (such as dosage, strength, duration)	Methods, section 2.3
	9c	Changes in therapeutic intervention (with rationale)	N/A
Follow-up and Outcomes	10a	Clinician and patient-assessed outcomes (if available)	Results
	10b	Important follow-up diagnostic and other test results	N/A
	10c	Intervention adherence and tolerability (How was this assessed?)	Results
	10d	Adverse and unanticipated events	N/A
Discussion	11a	A scientific discussion of the strengths AND limitations associated with this case report	Discussion
	11b	Discussion of the relevant medical literature **with references**.	Discussion
	11c	The scientific rationale for any conclusions (including assessment of possible causes)	Discussion
	11d	The primary "take-away" lessons of this case report (without references) in a one paragraph conclusion	Discussion
Patient Perspective	12	The patient should share their perspective in one to two paragraphs on the treatment(s) they received	Results, section 3.3
Informed Consent	13	Did the patient give informed consent? Please provide if requested	**Yes** ☑ **No** ☐

Appendix 2: Outcome measures and properties

Outcome Measure	ICF Level	Level of Measurement	Measurement Properties
Fugl-Meyer Assessment Upper Extremity (FMA-UE)	Body Function and Structures	Continuous (ratio)	Scored 0-66, higher = less upper limb impairment (Page et al., 2012).<br><br>Subsections: arm, wrist, hand, and coordination (Page et al., 2012).<br><br>MCID = 5.25 (Page et al., 2012).<br><br>Excellent criterion validity (r_s = 0.91-0.96) (Wei et al., 2011; See et al., 2013).<br><br>Excellent interrater and intrarater reliability (intraclass correlation coefficient (ICC) = 0.98-0.99) (Wei et al., 2011; See et al., 2013).
Action Research Arm Test (ARAT)	Activity	Continuous (ratio)	Scored 0-57, higher = greater arm activity/function (Yozbatiran et al., 2008).<br><br>Subsections: grasp, grip, pinch, and gross arm movement (Yozbatiran et al., 2008).<br><br>MCID = 5.7 (Van der Lee et al., 2001).<br><br>Excellent concurrent validity ($r = 0.94$, $p < 0.01$) (Yozbatiran et al., 2008).<br><br>Excellent interrater and intrarater reliability (ICC = 0.989-0.995) (Van der Lee et al., 2001).
Stroke Self-Efficacy Questionnaire (SSEQ)	Activity,<br><br>Participation	Continuous (ratio)	A 13-item self-reported scale of self-efficacy, items scored 0-10 (Riazi et al., 2014).<br><br>Total score 0-130, higher = greater self-efficacy (Riazi et al., 2014).<br><br>MCID = 3.3-3.7 (Wu et al., 2024).<br><br>Good internal consistency ($\alpha = 0.90$) (Jones et al., 2008).<br><br>High criterion validity ($r = 0.803$, $p < 0.001$) (Jones et al., 2008).

			High test-retest reliability (ICC = 0.86) (Jones et al., 2008).
EuroQol-5 Dimension Questionnaire (EQ-5D-5L) and EuroQol Visual Analogue Scale<br><br>(EQ-VAS)	Body Function and Structures, Participation	Continuous (interval for<br><br>EQ-5D-5L utility score, ratio for<br><br>EQ-VAS)	Health-related quality of life measures (Chen et al., 2016).<br><br>EQ-5D-5L<br><br>A 1-5 descriptive system of five dimensions: mobility, self-care, usual activities, pain/discomfort, and anxiety/depression (Chen et al., 2016). Results are converted to a utility score using Australian preference weights, higher utility score = better quality of life (Norman et al., 2013).<br><br>MCID = 0.10 (Chen et al., 2016).<br><br>Fair to good concurrent validity (r_s = 0.255-0.703) (Chen et al., 2016).<br><br>EQ-VAS<br><br>Scored 0-100, higher = better perceived health (Chen et al., 2016).<br><br>MCID = 8.61-10.82 (Chen et al., 2016).<br><br>Fair concurrent validity (r_s = 0.249-0.345) (Chen et al., 2016).

Appendix 3: Interview Guide

Interview Guide

Please note that this interview is being recorded and will be transcribed for analysis. Do you consent to this?

Qu 1: Tell me about your experience with Neuroboost, including the things you did and didn't like about the program?

Qu 2: Have you noticed any changes in your arm since completing Neuroboost? Please discuss.

Qu 3: Have you noticed any changes in your participation in activities since completing Neuroboost? Please discuss.

Qu 4: Do you feel like your quality of life has changed since completing Neuroboost? Please discuss.

Qu 5: Do you see rehabilitation programs like Neuroboost playing a role in your journey/future?

Qu 6: Would you like to share anything else about the program?

References

Chen, P., Lin, K.C., Liing, R.J., Wu, C.Y., Chen, C.L., and Chang, K.C. (2016). Validity, responsiveness, and minimal clinically important difference of EQ-5D-5L in stroke patients undergoing rehabilitation. *Quality of Life Research* 25**,** 1585-1596.

Jones, F., Partridge, C., and Reid, F. (2008). The Stroke Self-Efficacy Questionnaire: Measuring individual confidence in functional performance after stroke. *Journal of Clinical Nursing* 17**,** 244-252.

Norman, R., Cronin, P., and Viney, R. (2013). A pilot discrete choice experiment to explore preferences for EQ-5D-5L health states. *Applied health economics and health policy* 11**,** 287-298.

Page, S.J., Fulk, G.D., and Boyne, P. (2012). Clinically important differences for the upper-extremity Fugl-Meyer Scale in people with minimal to moderate impairment due to chronic stroke. *Physical Therapy* 92**,** 791-798.

Riazi, A., Aspden, T., and Jones, F. (2014). Stroke Self-Efficacy Questionnaire: A Rasch-refined measure of confidence post stroke. *Journal of rehabilitation medicine* 46**,** 406-412.

See, J., Dodakian, L., Chou, C., Chan, V., Mckenzie, A., Reinkensmeyer, D.J., and Cramer, S.C. (2013). A standardized approach to the Fugl-Meyer assessment and its implications for clinical trials. *Neurorehabilitation and neural repair* 27**,** 732-741.

Van Der Lee, J.H., Beckerman, H., Lankhorst, G.J., and Bouter, L.M. (2001). The responsiveness of the Action Research Arm Test and the Fugl-Meyer Assessment scale in chronic stroke patients. *Journal of Rehabilitation Medicine* 33**,** 110-113.

Wei, X.J., Tong, K.Y., and Hu, X.L. (2011). The responsiveness and correlation between Fugl-Meyer Assessment, Motor Status Scale, and the Action Research Arm Test in chronic stroke with upper-extremity rehabilitation robotic training. *International Journal of Rehabilitation Research* 34**,** 349-356.

Wu, S.Y., Li, Y.C., Chen, Y.W., Chen, C.L., Pan, H.C., Lin, K.C., and Lau, H.Y. (2024). Construct validity, responsiveness, minimal detectable change, and minimal clinically important difference of the Stroke Self-Efficacy Questionnaire in individuals receiving stroke rehabilitation. *Disability and Rehabilitation***,** 1-9.

Yozbatiran, N., Der-Yeghiaian, L., and Cramer, S.C. (2008). A standardized approach to performing the Action Research Arm Test. *Neurorehabilitation and neural repair* 22**,** 78-90.
